# Biological and Genetic Aspects of Donor-Recipient Matching in HSCT

**DOI:** 10.1155/2012/212593

**Published:** 2012-12-09

**Authors:** Andrzej Lange, Colette Raffoux, Bronwen Shaw

**Affiliations:** ^1^Institute of Immunology and Experimental Therapy, Polish Academy of Science, Wroclaw, Poland; ^2^National Bone Marrow Donor Registry, Lower Silesian Center for Cellular Transplantation, Wroclaw, Poland; ^3^International Research Group on Hematopoietic Cell Transplantation (IRGHET), Foundation Jean Dausset, Paris, France; ^4^Anthony Nolan Research Institute, Royal Free Hospital, London, UK; ^5^Section of Haemato-Oncology, Royal Marsden Hospital, Surrey, UK

Bone marrow transplantation is a routine clinical activity offering salvage therapy in a number of hematological diseases and inborn errors. There are two obstacles that may delay or even postpone this curable treatment approach. The first is a lack of matched family donors, which affects up to 75% of patients. In this situation a search for unrelated donors, if successfully completed, makes this approach feasible. HLA genes of five loci (A, B, C, DR, and DQ) are currently considered as a basis for matching. Each day brings information of new alleles. Genetic typing can lead to detection of diversity at the single nucleotide level. It ensures that a level of matching is achieved resulting in transplant success rates similar to those seen among siblings sharing the same HLA genotype. While we wish to have a perfect match, also important is elapsing time during the search process, which is related to the presence in the patient of rare alleles and unusual B-C, DR-DQ associations. Having a primary typing of a patient we can predict the chance for a proper match. Each day new donors are recruited worldwide. Iterative searching must be applied in the latter situation. In some cases 6 or more potential donors are required to have a donor accepted by a clinician. Finally, a compromise must be reached between the aspiration of matching at the level of 10 alleles and the urgency of transplantation in patients suffering from relapsing disease. To facilitate the decision-making process, modern information technology must be at hand. The search process includes the complete donor pool which is screened for potential donors. The chosen potential donors must be activated for confirmatory typing which includes 5 loci specificities typed at the high resolution level with exchange of information between registries and the hospital iteratively coming to the optimal decision. The process must be reliable, safe, and transparent, and must operate efficiently in real time. The European Marrow Donor Information System (EMDIS), used in many countries worldwide, ensures fulfillment of the above requirements. The present volume illustrates the above points, supporting the rational basis for the decision-making process.

Identification of HLA alleles in populations with a genetic background composed of different ancestral gene compositions may depict the prevalent component in the ethnicity. The latter can not only facilitate the search process but also provide some information on the presence of factors modifying the risk of graft-versus-host disease. You can read about that in this volume. Non-HLA genetic factors influencing the natural history of hematological malignancies and also shaping the risk of post-HSCT complications are being investigated by several groups of investigators. Among non-HLA genetic factors, probably killer immunoglobulin-like receptors (KIRs) and factors associated with the NOD2/CARD15 polymorphism have the best reputation for influencing the outcome of HSCT. Described and then consequently typed in alloHSCT patients KIR haplotypes associate with the ability of an individual to mount an immune response. Single-nucleotide polymorphism of the *NOD2* gene influences inflammatory response to the bacterial cell wall components that may induce advert effects. Both associations discussed in this volume document the significance of environmental factors including infections for the overt clinical manifestation of a primary alloreactive response. Indeed, in another paper in this volume, the associations between bacterial infections, *NOD2* gene mutation associated features, and the vigorous immune response involving a proinflammatory T cell subpopulation producing IL-17 in mounting graft-versus-host disease are shown and illustrated. The role of non-genetic factors, useful during the donor-recipient matching process, is described using the example of seropositivity against CMV in donors and recipients. The absence of CMV IgG antibodies in donors constitutes a risk factor of reactivation of this virus and influences the incidence of aGvHD of patients post HSCT. Therefore, biological factors modifying the outcome of HSCT may include HLA and non-HLA genetic variant associated features. 

Genetic diversity and non-genetic factors influence the outcome of HSCT, which renders the process of matching a very complex task. To facilitate the final decision it is important to recognize the presence of different factors and then to put them in an order depending on their weights. The present special issue of Bone Marrow Research presenting genetic and non-genetic factors affecting the outcome of HSCT may serve as a complementary tool in the decision-making process while choosing an optimal donor for a given patient. Each pair should be analyzed for the presence or absence of genetic traits or non-genetic characteristics which in a complementary fashion may influence the outcome of HSCT. We hope that the present volume contributes some additional information supporting this notion. 

*Andrzej Lange* *Colette Raffoux* *Bronwen Shaw*

